# Selective peptide–guided transcytosis enhances extracellular vesicle–mediated siRNA delivery across the blood–brain barrier

**DOI:** 10.1016/j.jbc.2025.110942

**Published:** 2025-11-13

**Authors:** Jingwen Fang, Lingzhu Zhang, Ye Wang, Menghan Chen, Yanqiu He, Chen-Yu Zhang, Yujing Zhang, Xiaohong Jiang, Jing Li

**Affiliations:** 1 Nanjing Drum Tower Hospital Centre of Molecular Diagnostic and Therapy, State Key Laboratory of Pharmaceutical Biotechnology, School of Life Sciences, Nanjing University, Jiangsu Engineering Research Centre for MicroRNA Biology and Biotechnology, NJU Advanced Institute of Life Sciences (NAILS), Nanjing, Jiangsu, China; 2 Research Unit of Extracellular RNA, Chinese Academy of Medical Sciences, Nanjing, Jiangsu, China

**Keywords:** extracellular vesicles, siRNA delivery, guide peptides, RVG, blood–brain barrier

## Abstract

Extracellular vesicles (EVs) have clinically emerged as promising biocompatible vesicles for delivering therapeutic siRNAs to the central nervous system. Among targeting strategies, the rabies virus glycoprotein (RVG) peptide is the most commonly used modification on the EV surface to enable efficient systemic delivery of EVs. Although RVG is widely believed to facilitate blood–brain barrier (BBB) through receptor interactions, the underlying mechanism remains indirect and equivocal. Similarly, cell-penetrating peptide (CPP) modifications have been used to enhance BBB transport of various vehicles, such as CPP.16, which improves the brain delivery efficiency of adeno-associated virus 9 capsids. However, whether CPP.16 retains its delivery efficacy when applied to EVs remains unclear, raising concerns about carrier-specific limitations. In this study, we investigate the mechanisms underlying the transcytosis and delivery efficiency of RVG- and CPP.16-modified small EVs (sEVs) loaded with siRNAs. Using an *in vitro* BBB model, we found that these modifications do not alter the internalization of siRNAs by endothelial cells. Instead, these modifications appear to divert sEVs and siRNAs into transcytotic pathways, enabling their release into abluminal cells and subsequent target gene silencing. Moreover, RVG-sEVs primarily interact with the receptor and are internalized *via* clathrin-mediated endocytosis, leading to more efficient BBB penetration compared with CPP.16-sEVs. Consistently, *in vivo* studies demonstrate that RVG-sEVs deliver siRNAs more efficiently to both neurons and astrocytes compared with unmodified or CPP.16-sEVs. Our findings support the clinical potential of BBB-targeting peptides and provide critical insights for the rational selection of guiding peptides in central nervous system drug delivery.

The incidence of central nervous system (CNS) disease has steadily increased over recent decades. RNA interference–based gene therapy holds great promise for treating a wide range of diseases, including those affecting the CNS (1, 2). Noninvasive systemic delivery of siRNA is particularly appealing, as it enables widespread siRNA distribution while avoiding the risks associated with intracranial administration (3). However, the lack of well-characterized siRNA carriers capable of crossing the blood–brain barrier (BBB) remains a major obstacle to clinical application of RNA interference–based therapies for CNS disorders.

The BBB is a highly selective barrier formed by continuous endothelial cells sealed by tight junctions (TJs) and devoid of fenestrations, supported by astrocytes, pericytes, and microglia on the abluminal side, which together maintain barrier integrity (4, 5). This cellular architecture restricts paracellular passage and is further reinforced by the inherently low rates of transcytosis in BBB endothelial cells compared with peripheral vasculature, significantly limiting molecular transport into the brain (4). Therapeutic strategies for crossing the BBB commonly exploit endogenous transcytosis mechanisms, including receptor-mediated transcytosis, adsorptive-mediated transcytosis (AMT), and carrier-mediated transcytosis (4, 6). Receptor-mediated transcytosis is initiated when ligands bind to specific luminal receptors expressed on the BBB, such as insulin and transferrin receptors (7, 8, 9). AMT, in contrast, relies on nonspecific electrostatic interactions between positively charged molecules and the negatively charged glycocalyx of endothelial cells (6). These interactions lead to clathrin-dependent endocytosis or caveolin (CAV)-mediated endocytosis, followed by the endocytic vesicles partitioning into the early endosome factions of endothelial cells, finally resulting in the fusion of vesicles with the abluminal membrane of BBB (6, 7, 10, 11).

Extracellular vesicles (EVs) are membrane-bound vesicles released by cells, mainly consisting of large EVs (also known as microvesicles) and small EVs (sEVs, also known as exosomes). EVs play a critical role in intercellular communication by transferring functional cargo, including mRNA, noncoding RNAs, and proteins. Over the past decade, sEVs have emerged as particularly promising vesicles for systemic siRNA delivery, owing to their low immunogenicity and high biocompatibility. To enhance brain targeting, sEV surfaces are often modified with peptides that exploit endogenous transcytosis pathways to traverse the BBB (12, 13). A widely explored approach involves incorporating the rabies virus glycoprotein (RVG) peptide—a 29-amino acid peptide derived from the RVG—into EVs or liposomes to facilitate the BBB permeability (14, 15, 16). RVG binds to nicotinic acetylcholine receptor (nAChR), conferring neurotropic properties and facilitating targeted delivery to the CNS (17, 18). While some early studies dismissed peripheral nerve retrograde transport as a BBB-penetrating mechanism (19), accumulating evidence supports a mechanism of direct transcytosis across the BBB through RVG–receptor interaction (18, 20). Despite this widely held belief, the precise identification of the RVG receptor on BBB endothelial cells, endocytic machinery, and subsequent intracellular fate and efficiency of RVG-mediated delivery remain unclear, posing a barrier to clinical translation. In parallel, cell-penetrating peptides (CPPs) have also shown potential for delivering therapeutics into the brain. CPPs are normally positively charged short peptides, which enhance their electrostatic interactions with negatively charged glycoproteins on the cell membrane, thereby promoting AMT and facilitating BBB penetration in a nonspecific manner (6, 21). CPP.16, a six-amino-acid peptide, has been engineered into adeno-associated virus 9 capsids to enhance BBB penetration (22). Yet, it remains unknown whether this peptide optimized for viral vectors retains efficacy when applied to EVs.

Given the therapeutic promise of sEV as a delivery tool, our study aims to address key knowledge gaps in peptide-guided siRNA delivery to the CNS *via* sEVs. We investigate and compare the roles of RVG and CPP.16 peptides in EV-mediated siRNA transport, dissect their mechanisms in transvascular transport, and evaluate their delivery efficiency in the brain. Our findings clarify the mechanistic basis for peptide-guided EV delivery and will inform the rational design of siRNA-based therapeutics for CNS disorders.

## Results

### Preparation of peptide-modified sEVs loaded with siRNAs

**Figure 1:**
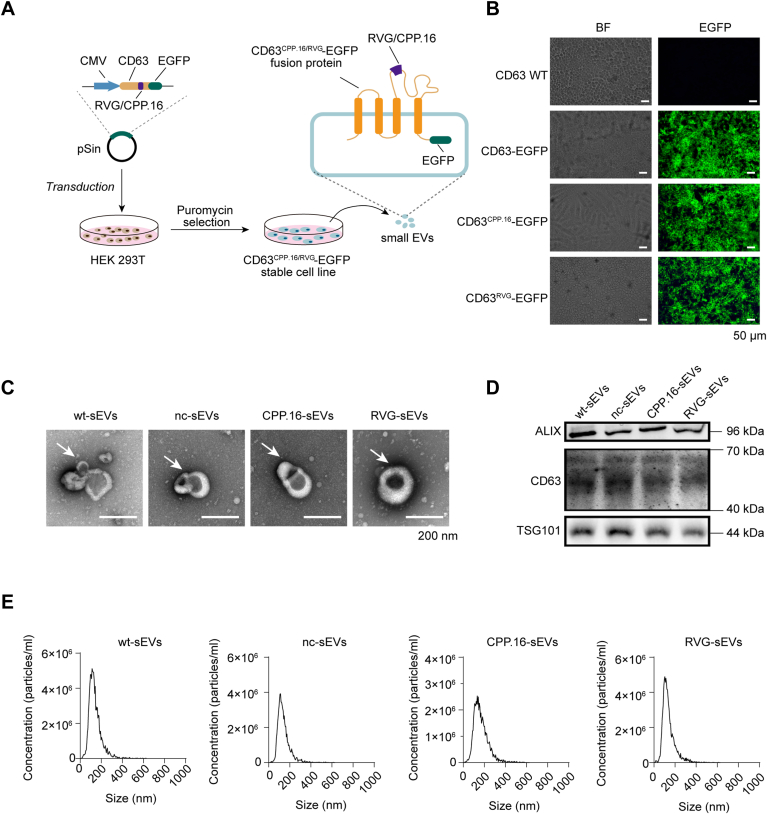
**Preparation and characterization of peptide-modified sEVs.***A,* schematic illustration of generation of HEK293T cell lines stably expressing CD63–EGFP, RVG–CD63–EGFP, or CPP.16–CD63–EGFP to produce modified sEVs. *B,* fluorescence images of engineered cell lines showing membrane-localized EGFP expression. The scale bar represents 50 μm. *C,* transmission electron microscopy (TEM) images of sEVs isolated from wt or engineered cell lines. The scale bar represents 200 nm. *D,* Western blot analysis of sEV markers (ALIX, CD63, and TSG101) in sEVs isolated from wt or engineered cell lines. *E,* nanoparticle tracking analysis (NTA) of sEVs showing a mean particle size of 120 ± 15 nm (*n* = 3 per group). See also Fig. S1; uncropped Western blot images for (*D*) are shown in Fig. S6. EGFP, enhanced GFP; HEK293T, human embryonic kidney 293T cell line; sEV, small extracellular vesicle.

To generate sEVs displaying RVG or CPP.16 peptides on their surface, the peptide sequences were genetically fused to the extracellular domain of CD63, a tetraspanin enriched in sEVs and used as a scaffold for surface peptide presentation and tag association (23). Enhanced GFP (EGFP) was appended to the C terminus of CD63 to facilitate sEV visualization. A CD63–EGFP fusion protein lacking any peptide was used as a negative control (nc). Lentivirus-encoding fusions of CD63–EGFP, CD63–RVG–EGFP, and CD63–CPP.16–EGFP were stably transduced into human embryonic kidney 293T (HEK293T) cells, enabling continuous secretion of engineered sEVs (Fig. 1*A*). Successful expression of all three constructs was confirmed by confocal microscopy, which showed strong EGFP fluorescence in transduced cells (Fig. 1*B*). sEVs derived from these stable cell lines, designated as nc-sEVs, RVG-sEVs, and CPP.16-sEVs, or derived from wt cells (wt-sEVs), were subsequently isolated and characterized. The morphology of the sEVs was not changed as determined by transmission electron microscopy (Fig. 1*C*), and the presence of sEV biomarkers was validated by Western blot (Fig. 1*D*). Nanoparticle tracking analysis revealed comparable size distributions and concentrations among the groups (Fig. 1*E*). These results indicate that peptide modifications did not affect the sEV size, morphology, and biogenesis. To load engineered sEVs with siRNAs, siRNAs were transfected into three CD63–peptide–EGFP expressing donor cell lines (Fig. S1*A*). Quantitative RT–PCR (qRT–PCR) analysis revealed that comparable levels of siRNAs were detected in the isolated sEVs, indicating similar loading efficiencies across all three sEV types (Fig. S1, *B* and *C*). To verify that these siRNAs were actively secreted *via* sEVs rather than originating from residual contamination in the culture medium, sEV release was inhibited with GW4869. Upon this treatment, siRNAs were not detectable in centrifugation preparations, confirming that the detected siRNAs were specifically loaded into and secreted through sEVs (Fig. S1*D*). Collectively, these findings confirm the successful generation of peptide-modified sEVs efficiently loaded with siRNAs, making them suitable for downstream functional studies.

### RVG-sEVs more efficiently deliver siRNAs across the endothelial cells of the BBB

To evaluate the ability of sEVs to penetrate the BBB, we established an *in vitro* BBB model using a noncontact coculture system (24, 25). Human cerebral microvascular endothelial cells (hCMEC/D3) were seeded in the upper chamber, whereas U87MG astrocytes were cultured in the lower chamber (Fig. S2*A*). Over a 10-day coculture period, the formation of TJs in hCMEC/D3 cells was assessed by measuring transepithelial/transendothelial electrical resistance, a widely used indicator of TJ integrity (Fig. S2*B*). In addition, passage of labeled dextran, used as an indicator of paracellular permeability, was reduced upon the formation of TJs (Fig. S2*C*). In parallel, expression of the TJ-associated protein zonula occludens-1 (ZO-1) progressively increased over time (Fig. S2*D*), further confirming the establishment of a functional BBB model.

**Figure 2:**
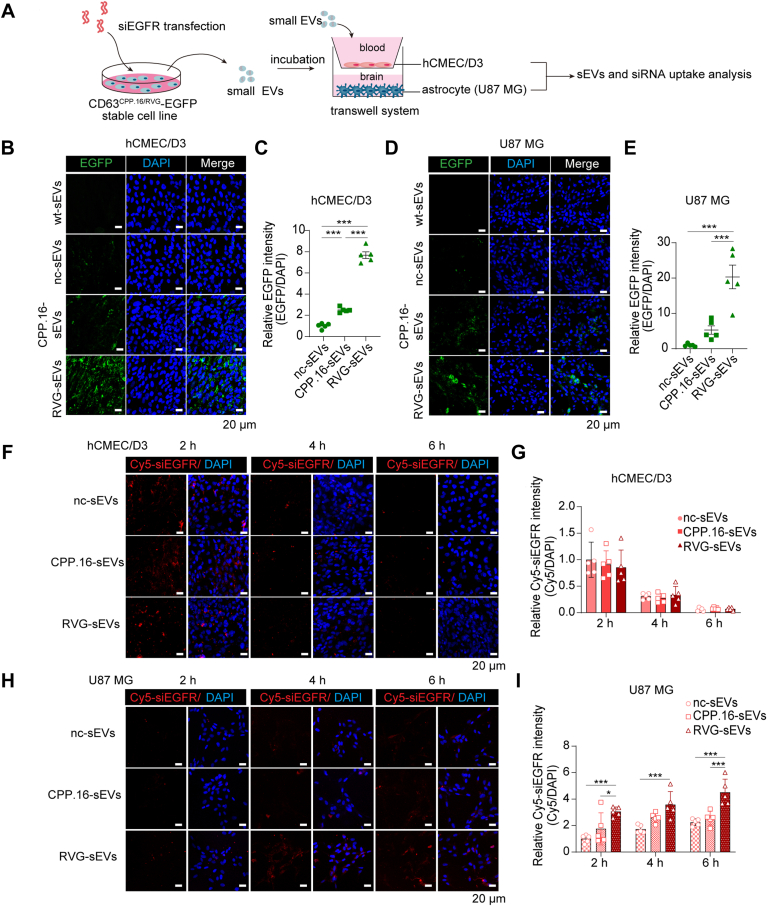
**RVG–sEVs enhance siRNA delivery across the BBB endothelial cells.***A,* experimental design: Engineered sEVs (nc-sEVs, CPP.16–sEVs, and RVG–sEVs) loaded with Cy5-siRNA targeting EGFR were incubated with hCMEC/D3 cells in a BBB model. *B,* fluorescence images of sEV-EGFP (*green*) uptake in hCMEC/D3 cells in a BBB model. *C,* quantification of sEV-EGFP fluorescence intensity in hCMEC/D3 cells normalized to DAPI (*n* = 5 per group). *D,* fluorescence images of sEV-EGFP (*green*) uptake in U87MG cells in a BBB model. *E,* quantification of sEV-EGFP fluorescence intensity in U87MG cells normalized to DAPI (*n* = 5 per group). *F,* fluorescence images of Cy5-siRNA *(red*) uptake in hCMEC/D3 cells after 2-, 4-, or 6-h incubation. Nuclei: DAPI (*blue*). The scale bar represents 20 μm. *G,* quantification of Cy5-siRNA fluorescence intensity in hCMEC/D3 cells normalized to DAPI (*n* = 5 per group). *H,* fluorescence images of Cy5-siRNA (*red*) uptake in U87MG cells after 2-, 4-, or 6-h incubation. Nuclei: DAPI (*blue*). The scale bar represents 20 μm. *I,* quantification of Cy5-siRNA fluorescence intensity in U87MG cells normalized to DAPI (*n* = 5 per group). Data are presented as the mean ± SEM. *p* Values were determined using one-way ANOVA in *C* and *E* and using two-way ANOVA followed by Tukey’s multiple comparison test in *G* and *I*, ∗*p* < 0.05, ∗∗∗*p* < 0.001. See also Figure S2. BBB, blood–brain barrier; CPP, cell-penetrating peptide; DAPI, 4′,6-diamidino-2-phenylindole; EGFP, enhanced GFP; EGFR, epidermal growth factor receptor; hCMEC, human cerebral microvascular endothelial cell; nc, negative control; sEV, small extracellular vesicle.

To assess transcytosis efficiency, nc-sEVs, CPP.16-sEVs, and RVG-sEVs were respectively applied to the upper chamber containing hCMEC/D3 cells (Fig. 2*A*). Importantly, sEV treatment did not disrupt TJ integrity, as evidenced by unchanged ZO-1 expression (Fig. S2*E*). Two hours postincubation, all three sEV types, visualized *via* EGFP fluorescence, were internalized by hCMEC/D3 cells. Notably, cells treated with RVG-sEVs exhibited significantly stronger EGFP signals compared with those treated with CPP.16-sEVs or nc-sEVs (Fig. 2, *B* and *C*). Six hours postincubation, sEVs were detected in glioblastoma (U87MG) cells seeded on the basal side of the BBB model, with RVG-sEVs showing the highest levels of accumulation (Fig. 2, *D* and *E*). These findings indicate that RVG modification significantly enhances the translocation of sEVs across the BBB, supporting its utility for brain-targeted siRNA delivery.

Next, the efficiency of siRNA delivery was evaluated. Equal amounts of Cy5-labeled siRNAs were loaded into the three sEV types and incubated with the hCMEC/D3 cells of the BBB model. Interestingly, comparable levels of Cy5-siRNAs were detected in hCMEC/D3 cells treated with nc-sEVs, CPP.16-sEVs, or RVG-sEVs at 2, 4, and 6 h postincubation, suggesting that peptide modifications did not enhance siRNA uptake by endothelial cells as initially expected (Fig. 2, *F* and *G*). A decline in intracellular siRNA levels was observed at 4 h relative to 2 h postincubation (Fig. 2, *F*, *second lane*, and *G*), reflecting the siRNA degradation or transcytosis. Strikingly, Cy5 siRNA signals were significantly increased in U87MG cells at 4 h post treatment with RVG-sEVs, whereas siRNAs delivered by nc-sEVs or CPP.16-sEVs were scarcely detected in basal layer cells (Fig. 2, *H* and *I*). Consistent with this, qRT–PCR analysis showed similar levels of siRNA uptake by endothelial cells across all groups (Fig. S2F), whereas RVG-sEVs transferred significantly more siRNAs to the basal layer of U87MG cells (Fig. S2*G*), leading to a more effective reduction in the expression of the target gene, epidermal growth factor receptor (EGFR), in the basal layer of U87MG cells compared with CPP.16-sEVs and nc-sEVs (Fig. S2*H*).

These results suggested that, while peptide modification does not improve siRNA uptake by endothelial cells, RVG-sEVs markedly enhance the transcytosis of siRNAs across the BBB, enabling efficient delivery to brain parenchymal cells.

### RVG-sEVs undergo more efficient transcytosis across BBB endothelial cells

**Figure 3:**
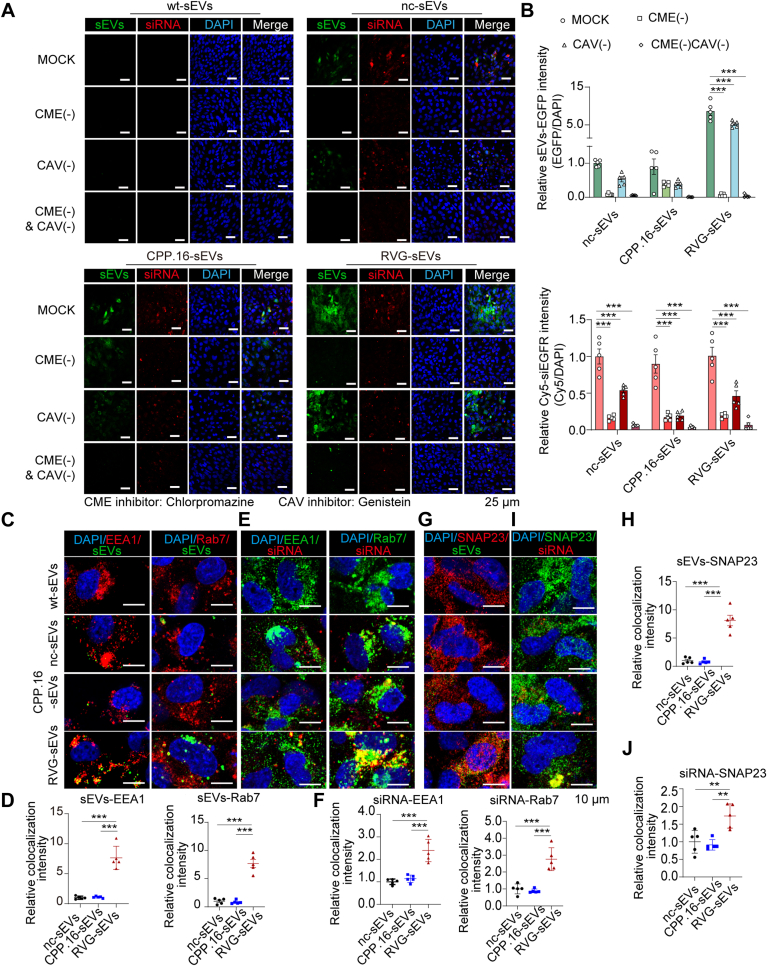
**RVG-sEV transcytosis efficiency in BBB endothelial cells.***A,* fluorescence images of sEV-EGFP (*green*) and Cy5-siRNA (*red*) in hCMEC/D3 cells treated with chlorpromazine (CME inhibitor, 20 μM, 30 min) or genistein (CAV inhibitor, 200 μM, 30 min). Nuclei: DAPI (*blue*). The scale bar represents 25 μm. *B,* quantification of sEV-EGFP (*upper panel*) and Cy5-siRNA (l*ower panel*) fluorescence intensity normalized to DAPI (*n* = 5 per group). *C,* fluorescence images showing colocalization of sEV-EGFP (*green*) with early endosome marker EEA1 *(red*, *left*) or late endosome marker Rab7 *(red*, *right*). Nuclei: DAPI (*blue*). The scale bar represents 10 μm. Individual channels are shown in Fig. S3*A* and S3B. *D,* quantification of colocalization of sEV-EGFP with EEA1 or Rab7 normalized to DAPI (*n* = 5 per group). *E,* fluorescence images showing colocalization of Cy5-siRNA (*red*) with EEA1 (*green*, *left*) or Rab7 (*green*, *right*). Nuclei: DAPI (*blue*). The scale bar represents 10 μm. Individual channels are shown in Fig. S3, *A* and *B*. *F,* quantification of colocalization of Cy5-siRNA with EEA1 or Rab7 normalized to DAPI (*n* = 5 per group). *G,* fluorescence images showing colocalization of sEV-EGFP (*green*) with SNAP23 (*red*). Nuclei: DAPI (*blue*). The scale bar represents 10 μm. Individual channels are shown in Fig. S3*C*. *H,* quantification of colocalization of sEV-EGFP with SNAP23 normalized to DAPI (*n* = 5 per group). *I,* fluorescence images showing colocalization of Cy5-siRNA (*red*) with SNAP23 (*green*). Nuclei: DAPI (*blue*). The scale bar represents 10 μm. Individual channels are shown in Fig. S3*C*. *J,* quantification of colocalization of siRNA with SNAP23 normalized to DAPI (*n* = 5 per group). Data are presented as the mean ± SEM. *p* Values were determined using two-way ANOVA in *B* and one-way ANOVA followed by Tukey’s multiple comparison test in *D*, *F*, *H*, and *J*, ∗∗*p* < 0.01, ∗∗∗*p* < 0.001. See also Fig. S3. BBB, blood–brain barrier; CAV, caveolin; DAPI, 4′,6-diamidino-2-phenylindole; EEA1, early endosome antigen 1; hCMEC, human cerebral microvascular endothelial cell; RVG, rabies virus glycoprotein; sEV, small extracellular vesicle.

Next, we investigated the transcytosis of peptide-modified sEVs using our *in vitro* BBB model. Transcytosis across the endothelial cells of the BBB typically begins with clathrin-mediated endocytosis (CME) or CAV-mediated endocytosis at the apical membrane of brain microvascular endothelial cells, followed by intracellular trafficking and eventual release at the basolateral membrane. To identify the specific endocytic pathways involved, CME and CAV-1-mediated endocytosis were selectively inhibited in hCMEC/D3 cells during sEV incubation. Inhibition of CME (−) significantly reduced the internalization of both RVG-sEVs and CPP.16-sEVs as well as their associated siRNAs (Fig. 3, *A* and *B*). In contrast, inhibition of CAV-1-mediated endocytosis (CAV [−]) impaired the uptake of CPP.16-sEVs and their siRNAs but had little effect on RVG-sEVs (Fig. 3, *A* and *B*). These results suggest that RVG-sEVs primarily utilize CME, whereas CPP.16-sEVs engage both CME and CAV-mediated endocytosis.

To further examine intracellular trafficking, we assessed the colocalization of sEVs and siRNAs with early endosome antigen 1 (EEA1), a marker of early endosomes, and Rab7, a marker of late endosomes. RVG-sEVs (Fig. 3, *C* and *D*) and their siRNA cargo (Fig. 3, *E* and *F*) showed strong colocalization with both EEA1 and Rab7, whereas CPP.16-sEVs, nc-sEVs, and their associated siRNAs exhibited minimal colocalization with these markers (Fig. 3, *C*–*F*). These findings indicate that RVG-sEVs and their siRNAs are more efficiently trafficked through the endosomal system compared with other sEV types. Finally, we examined the colocalization of sEVs and their siRNA cargos with soluble NSF attachment protein (SNAP23), a marker involved in endosome fusion with the basolateral membrane. Significant colocalization of RVG-sEVs (Fig. 3, *G* and *H*) and their siRNAs (Fig. 3, *I* and *J*) with SNAP23 was observed, suggesting that RVG-sEVs and their cargos are efficiently released into the abluminal compartment.

Collectively, these results demonstrate that RVG peptides enhance sEV transcytosis by facilitating CME, efficient endosomal trafficking, and basolateral release, thereby facilitating siRNA delivery across the BBB into the brain parenchyma.

### The nAChR and p75 neurotrophic receptor mediate the transcytosis of RVG-sEVs across the BBB

The nAChR is a ligand-gated ion channel activated by acetylcholine or nicotine binding. The p75 neurotrophic receptor (p75NTR), also known as CD271 or the low-affinity nerve growth factor (NGF) receptor, is a key modulator of neurotropin signaling. Rabies virus has been reported to enter neurons *via* receptor-mediated endocytosis through interactions with nAChR or p75NTR. Notably, both receptors are also expressed on brain endothelial cells, supporting a widely held but previously unproven hypothesis that they facilitate the transcytosis of RVG-modified vesicles across the BBB.

**Figure 4:**
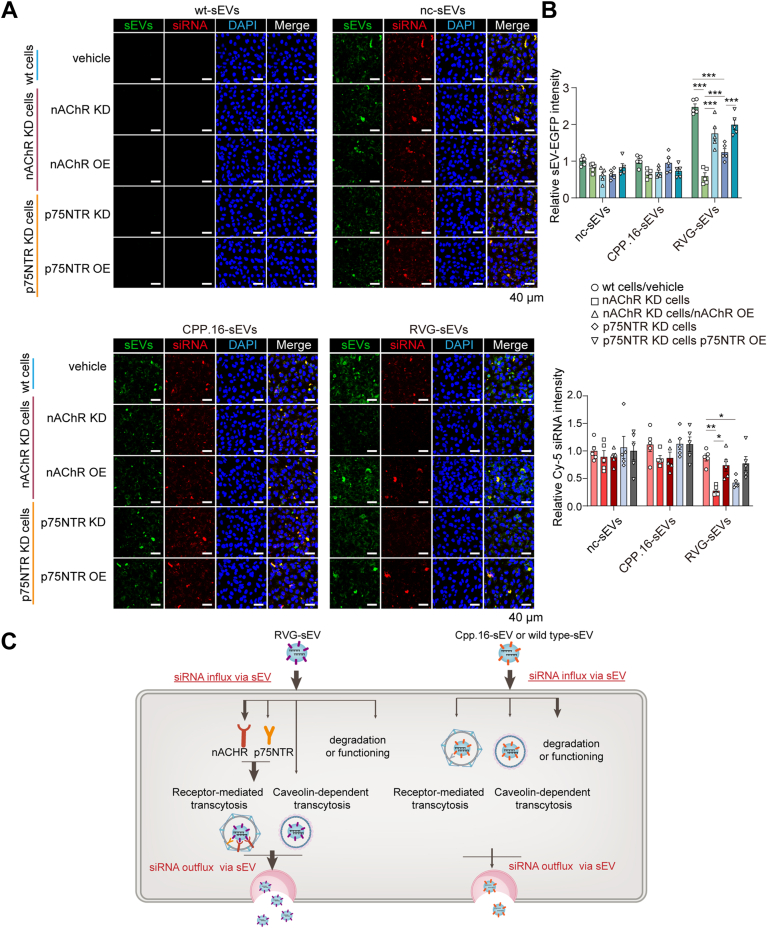
**nAChR and p75NTR mediate RVG-sEV transcytosis.***A,* fluorescence images of sEV-EGFP (*green*) and Cy5-siRNA (*red*) in wt hCMEC/D3 cells, nAChR KD cells, nAChR-rescued cells (nAChR OE), p75NTR KD cells, or p75NTR-rescued cells (p75NTR OE). Nuclei: DAPI (*blue*). The scale bar represents 40 μm. *B,* fluorescence intensity quantification of sEV-EGFP and Cy5-siRNA normalized to DAPI. (*n* = 5 per group). *C,* schematic representation of nc-sEVs, CPP.16-sEVs, and RVG-sEVs transcytosed by hCMEC/D3 cells. Representative results are presented as the mean ± SEM. *p* Values are calculated using two-way ANOVA followed by Tukey’s multiple comparisons test in *B*, ∗*p* < 0.05, ∗∗*p* < 0.01, ∗∗∗*p* < 0.001, See also Fig. S4. DAPI, 4′,6-diamidino-2-phenylindole; EGFP, enhanced GFP; hCMEC, human cerebral microvascular endothelial cell; KD, knockdown; nAChR, nicotinic acetylcholine receptor; nc, negative control; p75NTR, p75 neurotrophic receptor; RVG, rabies virus glycoprotein; sEV, small extracellular vesicle.

To evaluate the roles of nAChR and p75NTR in RVG-sEV internalization, we generated stable hCMEC/D3 cell lines with targeted knockdown of either receptor. The most effective siRNA sequences were selected based on knockdown efficiency and used to establish the cell lines (Fig. S4, *A* and *B*). Receptor expression was markedly reduced without affecting BBB integrity (Fig. S4, *C*–*F*). Knockdown of either receptor significantly impaired RVG-sEV and siRNA uptake, with nAChR knockdown producing a stronger effect (Fig. 4, *A* and *B*). Crucially, rescuing the respective receptors (Fig. S4, *C* and *D*) restored RVG-sEV and siRNA uptake, confirming the specificity of receptor-mediated endocytosis (Fig. 4*A*). In contrast, uptake of CPP.16-sEVs, modified with a positively charged peptide and internalized primarily through nonspecific adsorptive-mediated endocytosis, was unaffected by knockdown of either receptor. Consistent results were obtained when these receptors were pharmacologically inhibited with their respective competitive ligands, benzethonium chloride, and NGF (Fig. S4*G*). These results demonstrate that RVG-sEVs are internalized *via* both nAChR- and p75NTR-mediated pathways, with nAChR playing the dominant role in facilitating endocytosis across the BBB.

In summary, these data suggest that RVG modification primarily promotes the transcytotic trafficking of siRNAs. Although RVG-modified sEVs exhibited stronger intracellular fluorescence signals in endothelial cells, the total amount of internalized siRNAs was comparable across all groups, suggesting a divergence in the fate of internalized agents. We speculate that RVG-modified sEVs may be less prone to lysosomal degradation, resulting in enhanced intracellular stability and fluorescence retention. The detected siRNAs likely reflect molecules transiting through the transcytotic pathway or functionally retained in the cytosol for gene silencing. Thus, rather than increasing total cellular uptake, RVG modification appears to enhance the entry of internalized siRNAs into the transcytotic route (Fig. 4*C*).

### RVG peptide enhances targeted delivery of sEVs and siRNAs to neurons and astrocytes *in vivo*

**Figure 5:**
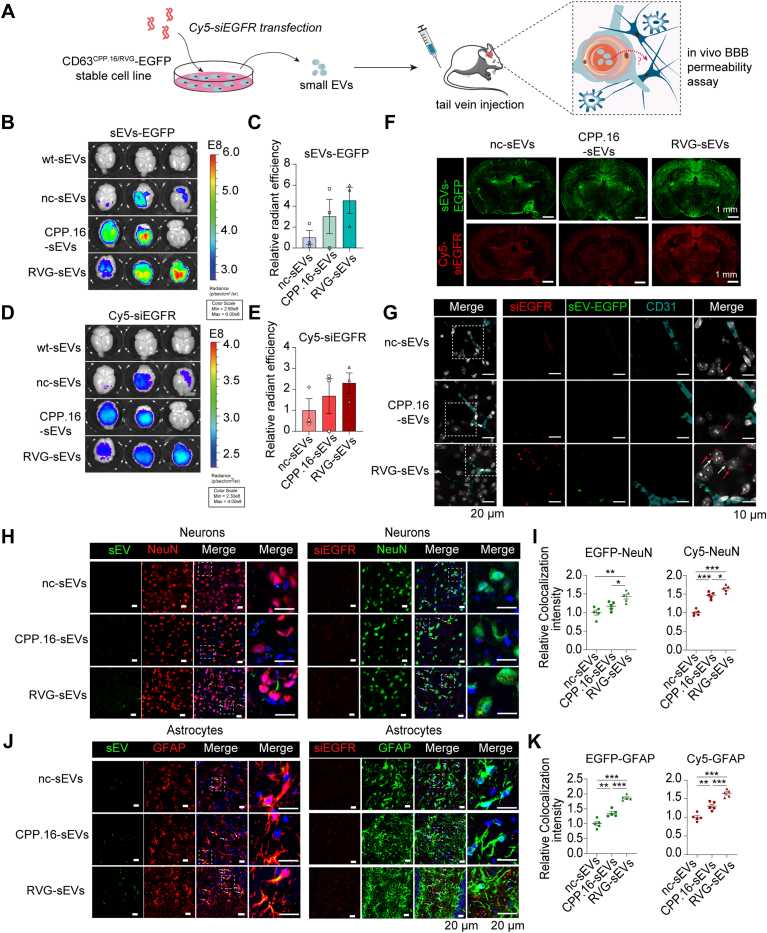
**RVG-sEVs achieve targeted *in vivo* brain delivery.***A,* experimental design for assessing biodistribution and penetration of engineered sEVs (nc-sEVs, RVG-sEVs, and CPP.16-sEVs) loaded with siRNA targeting EGFR in mice. *B, in vivo* imaging system (IVIS) analysis of sEV-EGFP in the mouse brains following intravenous injection of engineered sEVs loaded with Cy5-siRNA. Excitation/emission wavelengths: 480/560 nm (sEV-EGFP) and 640/670 nm (Cy5-siRNA). *C,* quantification of sEV-EGFP intensity in the mouse brain (*n* = 3 per group). *D,* IVIS analysis of Cy5-siRNA in the mouse brains. *E,* quantification of Cy5-siRNA intensity in mouse brain (*n* = 3 per group). *F,* whole-brain fluorescence images showing distribution of sEV-EGFP (*green*) and Cy5-siRNAs (*red*). The scale bar represents 1 mm. *G,* fluorescence images of sEV-EGFP *(green*) and Cy5-siRNA (*red*) in the brain parenchyma. CD31 (*cyan*) was used to indicate brain blood vessels. Nuclei: DAPI (*white*). *Arrows* indicate representative sEVs (*white*) and siRNA (*red*) signals outside vessels. The scale bar represents 20 μm; zoom-in, 10 μm. *H,* fluorescence images of sEV-EGFP (*left*) and Cy5-siRNA *(right*) colocalization with neurons (NeuN^+^). Nuclei: DAPI (*blue*). The scale bar represents 20 μm; zoom-in, 20 μm. *I,* quantification of sEV-EGFP (*left*) and Cy5-siRNA (*right*) colocalization with neurons normalized to DAPI (*n* = 5 per group). *J,* fluorescence images of sEV-EGFP (*left*) and Cy5-siRNA (*right*) colocalization with astrocytes (GFAP^+^). Nuclei: DAPI (*blue*). The scale bar represents 20 μm, zoom-in, 20 μm. *K,* quantification of sEV-EGFP (*left*) and Cy5-siRNA (*right*) colocalization with astrocytes normalized to DAPI (*n* = 5 per group). Data are presented as the mean ± SEM. *p* Values were determined using one-way ANOVA followed by Tukey’s multiple comparison test in *C*, *E*, *I*, and *K*, ∗*p* < 0.05, ∗∗*p* < 0.01, and ∗∗∗*p* < 0.001. See also Fig. S5. CPP, cell-penetrating peptide; DAPI, 4′,6-diamidino-2-phenylindole; EGFR, epidermal growth factor receptor; nc, negative control; RVG, rabies virus glycoprotein; sEV, small extracellular vesicle.

To evaluate the ability of RVG and CPP.16 peptides to mediate siRNA delivery to brain cells *in vivo*, we intravenously administrated sEVs loaded with Cy5-labeled siRNAs into mice (Fig. 5*A*). Brain tissues were harvested and analyzed using *in vivo* fluorescence imaging. Although all three types of sEVs were detected in the brain, RVG-sEVs (Fig. 5, *B*, *C* and *F*) and their siRNA cargo (Fig. 5, *D*–*F*) showed moderately higher overall accumulation. To precisely localize sEVs and siRNAs within the brain, we assessed their fluorescence distribution and histological localization. CD31 staining was used to demarcate brain vasculature. RVG-sEVs and their siRNA cargo were clearly observed beyond the vasculature, within the brain parenchyma, whereas CPP.16-sEVs and nc-sEVs were largely retained in proximity to blood vessels (Fig. 5*G*). The EGFR expression was more significantly inhibited in the brain of mice administrated RVG-sEVs (Fig. S5*A*). These results suggest that RVG modification enhances the transverse of sEVs across the BBB and entry into the parenchymal space. Within the parenchyma, colocalization analysis revealed that RVG-sEVs and their siRNAs were internalized by both neurons and astrocytes, as evidenced by overlap with NeuN (a neuronal marker; Fig. 5, *H* and *I*) and GFAP (an astrocyte marker; Fig. 5, *J* and *K*). Collectively, these findings demonstrate that the RVG peptide significantly improves the targeted delivery of siRNA-loaded sEVs to diverse brain cell populations *in vivo*.

## Discussion

RVG and CPP.16 are used to enhance the delivery of different carriers into the brain. However, the mechanisms by which these CPPs mediate BBB translocation remain indirect and poorly defined. In this study, we aimed to systematically elucidate the complete process of CPP-mediated BBB crossing.

RVG peptide is the most utilized CPP for CNS delivery. In the earliest applications, a chimeric RVG peptide consisting of 29 amino acids was directly conjugated to siRNA (20). To enhance siRNA binding, a nonamer of arginine residues was added to the C terminus of the peptide (26). RVG-mediated delivery into the brain parenchyma includes sequential steps: (1) transcytosis across the endothelial cells of the BBB, initiated by the RVG peptide, which facilitates the transport of RVG-modified carriers or therapeutics into the CNS and (2) subsequent uptake by brain parenchymal cells following BBB penetration. While the neurotropism of RVG has been attributed to its binding with nAChRs expressed on neural cells (20), this interaction alone does not fully explain its ability to mediate transport across the BBB. Previous studies have suggested the plausibility of RVG-triggered transcytosis, implicating receptor-mediated mechanisms involving surface proteins, such as nAChR (27) and p75NTR (28) on the brain capillary endothelial cells. However, direct evidence of how RVG facilitates endothelial transcytosis has remained elusive, leaving a critical gap in our understanding of its role in CNS-targeted delivery.

In this study, we reveal the detailed mechanism and machinery of RVG-initiated transcytosis across the endothelial cells of the BBB by binding with nAChR and p75NTR. Notably, inhibition of nAChR led to a more pronounced reduction in RVG-sEV uptake than inhibition of p75NTR, suggesting that nAChR serves as the primary receptor mediating RVG-induced endocytosis. This may be attributed either to a lower binding affinity of RVG for p75NTR or to higher expression levels of nAChR on the surface of brain endothelial cells (29). It is known that endocytosis plays a central role in regulating membrane-bound nAChR levels and, consequently, synaptic function (30). Unlike G-protein–coupled receptors, the intracellular trafficking pathways of ionotropic receptors like nAChR are not yet well characterized. Previous reports have shown that both agonists (*e.g.*, carbachol and nicotine) and antagonists (*e.g.*, α-bungarotoxin) can induce the endocytosis of nAChRs (31). Therefore, RVG, another exogenous antagonist, can also initiate endocytosis.

In our study, CPP.16 does not enhance the transport of EV and their siRNAs into the brain as efficiently as RVG, albeit CPP.16 has previously been reported to enhance the transvascular delivery of adeno-associated virus 9 particles (22). The cellular uptake mechanism of CPPs depends on a variety of factors. In addition to endocytosis, some CPPs directly penetrate into the cytosol through pore formation and destabilization of the cell membrane (32). Direct penetration primarily transports the cargo to the cell cytoplasm (33), in which EV and siRNA may directly function in the cytoplasm, therefore not favoring efficient transcytosis. The disparity in transport efficiency implies potential caveats in the crossutilization of targeting peptides across different delivery platforms, underscoring the need for carrier-specific optimization when engineering peptide-functionalized delivery systems.

Interestingly, as shown in Figure 2, modification of sEVs with targeting peptides did not appear to affect the overall delivery of siRNAs into endothelial cells within the *in vitro* BBB model. This suggests that RVG-initiated endocytosis does not necessarily enhance the intracellular accumulation of siRNAs in the endothelial cells. Once internalized, siRNAs within endothelial cells can follow one of the three major fates: (1) degradation in lysosomes, (2) transcytosis across the cell, or (3) release into the cytosol, where they may exert functional activity (32). Therefore, comparable intracellular siRNA levels across treatment groups may mask differences in downstream delivery outcomes. Only siRNAs that successfully undergo transcytosis can be efficiently transferred into the brain parenchyma.

Our findings indicate that RVG modification primarily promotes the transcytotic trafficking of siRNAs, thereby enhancing their release into the brain, rather than increasing total cellular uptake by endothelial cells. Although RVG-modified sEVs exhibited stronger fluorescence signals within endothelial cells, this did not correspond to an increase in siRNA content, suggesting a divergence in the fate of internalized cargo. We speculate that RVG-modified sEVs may be less prone to lysosomal degradation, resulting in greater intracellular stability and fluorescence, whereas the detected siRNAs likely include those either in transit *via* transcytosis or retained functionally within the cytosol.

## Experimental procedures

### Cells, reagents, and antibodies

The HEK293T cell line, the human cortical microvascular endothelial cell line hCMEC/D3, and the glioblastoma cell line U87MG were purchased from the Institute of Biochemistry and Cell Biology at the Shanghai Institute for Biological Science at the Chinese Academy of Sciences. HEK293T cells and U87MG cells were maintained in Dulbecco’s modified Eagle’s medium (Gibco) supplemented with 10% fetal bovine serum (FBS; Gibco) and 1% penicillin–streptomycin (Gibco). hCMEC/D3 cells were maintained in endothelial cell medium (ScienCell) supplemented with 10% FBS (ScienCell) and 1% penicillin–streptomycin (Gibco). All cells were cultured in a 5% CO_2_, water-saturated atmosphere. Short tandem repeat profiling and mycoplasma contamination testing were performed to authenticate all cell lines.

The following antibodies were used in this study: anti-nAChR, anti-p75NTR, anti-EGFP, anti-GFAP, anti-NeuN, and anti-CD31 antibodies were purchased from Abcam, and the anti-ZO1, anti-EEA1, anti-Rab7, anti-CD63, anti-TSG101, anti-Alix antibodies, horseradish peroxidase–conjugated Goat Anti-Mouse IgG (H + L), and horseradish peroxidase–conjugated Goat Anti-Rabbit IgG (H + L) were obtained from Proteintech; anti-SNAP23 antibody was purchased from Santa Cruz Biotechnology. Fluorescent secondary antibodies, including Alexa Fluor 546, Alexa Fluor 594, Alexa Fluor 647, Alexa Fluor 555, and Alexa Fluor 488, were purchased from Invitrogen.

### Animals

Six-week-old male C57BL/6J mice were purchased from GemPharmatech. The animals were housed under specific pathogen-free conditions and maintained in a temperature-controlled room with a 12-h light–dark cycle. All animal experiments were reviewed and approved by the Animal Ethical and Welfare Committee of Nanjing University (IACUC-2301001-1). Animal studies were designed and conducted following the ARRIVE (Animal Research: Reporting of *In Vivo* Experiments) guidelines.

### Design and assembly of plasmid constructs

Plasmids encoding CD63 fused with guide peptides (RVG or CPP.16) were generated as previously described (23). DNA sequences corresponding to RVG (YTIWMPENPRPGTPCDIFTNSRGKRASNG) or CPP.16 (TVSALK) were synthesized by GeneScript and inserted into the extracellular loop of CD63, immediately after the 147th amino acid residue. To enable visualization of EV, EGFP was fused to the N terminus of CD63 (34). The resulting fusion constructs (CD63–peptide–EGFP) were cloned into the pSin vector (Addgene) using the EcoRI and MluI restriction sites under the control of a cytomegalovirus promoter.

For gene knockdown, siRNA oligonucleotides targeting nAChR or p75NTR were cloned into the pLKO.1 vector (Addgene) using AgeI and EcoRI restriction sites under the control of the U6 promoter. For overexpression, the coding sequences of nAChR and p75NTR were inserted into the BamHI–EcoRI sites of the pcDNA3.1(+) vector (Thermo Fisher Scientific). All plasmids were designed and synthesized by GeneScript. The siRNA sequences are listed in Table S1.

### Lentivirus construction and generation of stable cell lines

Plasmids encoding CD63 fusion proteins or siRNAs targeting nAChR and p75NTR were cotransfected into HEK293T cells along with the packaging plasmids psPAX2 and pMD2.G at a molar ratio of 10:5:1 using Lipofectamine 2000 (Invitrogen). Lentiviral particles were harvested from the culture supernatant 48 h post-transfection, filtered, and used to infect target cells.

To generate cells stably expressing CD63 fusion proteins, HEK293T cells were seeded in 6-well plates and incubated with the corresponding lentiviral supernatant in the presence of 4 μg/ml polybrene (Yeasen). After 48 h, the medium was replaced with fresh culture medium. Infected cells were cultured as a polyclonal population under selection with 1 μg/ml puromycin (Beyotime). For monoclonal selection, the puromycin-resistant population was serially diluted and seeded into 96-well plates. After 14 days, EGFP-positive single-cell clones were identified under a fluorescence microscope and expanded for further use.

To generate hCMEC/D3 cells with nAChR and p75NTR knockdown, cells were infected with the corresponding lentivirus under the same transduction conditions. Infected cells were maintained as a polyclonal population under selection with 1 μg/ml puromycin (Beyotime).

### Transfection of fluorescent-labeled siRNAs

To load siRNA into sEVs, monoclonal HEK293T cells stably expressing CD63 fusion proteins were transfected with Cy5-labeled siRNA (Genescript) using Lipofectamine 2000 as previously described (35, 36). Briefly, cells were seeded in 10 cm dishes and cultured overnight. Cy5-labeled siRNAs (900 pmol) were incubated with cells in Opti-MEM. After 6 h of incubation, the transfection medium was removed, and the cells were washed several times with PBS to eliminate residual extracellular siRNAs. Fresh culture medium supplemented with 10% EV-depleted FBS was then added, and cells were incubated for an additional 24 h. The conditioned medium was subsequently collected for sEV isolation. And the transfected siRNA sequences (siEGFR) are listed in Table S1.

### sEV isolation and characterization

HEK293T stable cells transfected with siRNAs were cultured in sEV-depleted medium for 24 h. Conditioned medium was collected and sequentially centrifuged at 800*g* for 10 min to remove dead cells, followed by 3000*g* for 20 min to remove cellular debris. The resulting supernatant was further centrifuged at 10,000*g* for 30 min to remove large EVs. To isolate sEVs, the clarified supernatant was mixed with Total Exosomes Isolation Reagent (Vazyme) at a 3:1 (v/v) ratio and incubated overnight at 4 °C. The mixture was then centrifuged at 10,000*g* for 1 h at 4 °C. The resulting pellet was resuspended in PBS for subsequent analysis.

The particle size distribution and concentration were revealed by nanoparticle tracking analysis using a ZetaView PMX 110 system (Particle Metrix). Data were analyzed using ZetaVie software, version 8.0. sEV morphology was examined by transmission electron microscopy using an FEI Tecnai G2 Spirit operated at 120 kV (Thermo Fisher Scientific). The presence of established sEV markers, including CD63, TSG101, and Alix, was confirmed by Western blot analysis.

### Establishment of the *in vitro* BBB model

A noncontact *in vitro* BBB model was established using a 24-well Transwell system with a polycarbonate porous membrane (pore size: 0.4 μm; Biofil) as previously described (24, 25). hCMEC/D3 cells were seeded on the apical side of the insert membrane, whereas U87MG human glioblastoma cells were cultured in the basolateral (lower) chamber. The transendothelial electrical resistance was measured daily using a Millicell ERS-2 epithelial volt-ohm meter (Millipore) to assess the formation and integrity of the endothelial barrier. For paracellular permeability evaluation using dextrans, hCMEC/D3 cells seeded on transwell inserts were treated with Cy3-dextrans (70 kDa; Beyotime). Culture medium was collected for over 60 min and replaced with an equal volume of fresh media.

### *In vitro* and *in vivo* treatment of siRNA-loaded sEVs

For *in vitro* analysis of siRNA levels, hCMEC/D3 cells in the *in vitro* BBB model were incubated with 1.3 × 10^9^ sEVs (8 μg protein content) per well. At designated time points (2, 4, and 6 h), hCMEC/D3 cells and U87MG cells from the bottom chamber were collected to quantify the uptake of sEVs and siRNAs. For colocalization analysis of sEVs and siRNAs with endocytotic markers, cells were incubated with sEVs for 5 min (for EEA1), 15 min (for Rab7), and 20 min (for Snap23), followed by immunofluorescence staining and imaging.

For *in vivo* analysis, 3.2 × 10^9^ (20 μg protein content) sEVs were injected into mice *via* tail vein injection.

### Blockade of endocytosis and receptor–peptide interactions

hCMEC/D3 cells were pretreated with pharmacological inhibitors to block endocytic pathways or peptide–receptor interactions prior to sEV treatment. For inhibition of CME, cells were treated with 20 μM chlorpromazine (Anpel) for 30 min. CAV-mediated endocytosis was inhibited using 200 μM genistein (Meryer) under the same conditions.

To genetically block peptide–receptor interactions, hCMEC/D3 cell lines with stable knockdown of p75NTR and nAChR were generated. The detailed methodology for generating these stable cell lines is described in the corresponding section.

To block p75NTR–peptide interactions pharmacologically, cells were pretreated with 50 ng/ml NGF (Novoprotein) for 30 min. To block nAChR interactions, cells were treated with 42 benzethonium chloride (TargetMol) for 1 h. Cells were incubated with the indicated sEVs for 2 h, after which they were collected for analysis of sEV and siRNA uptake.

### Quantitative RT–PCR assay

Total RNA was extracted from cultured cells using TRIzol Reagent (Takara) according to the manufacturer’s instructions. Quantification of siRNAs was performed using a customized TaqMan MicroRNA assay (Applied Biosystems). Briefly, 1 μg of total RNA was reverse transcribed using AMV reverse transcriptase (Takara) and specific RT-primer (Applied Biosystems). qRT–PCR was performed on an ABI 7300 system (Applied Biosystems). For absolute quantification of siRNA levels, synthetic siRNAs were serially diluted and assessed using qRT–PCR to generate a standard curve. siRNA concentrations were calculated by referring to the standard curve and presented as the absolute amounts of siRNA in 1 μg total RNA (pmol/μg total RNA).

### Western blotting

Total protein from cells or sEVs was extracted using radioimmunoprecipitation assay lysis buffer (Beyotime). Protein concentrations were determined with a BCA assay kit (Vazyme). Lysates were used for electrophoresis on SDS-PAGE (10%) gel and transferred to polyvinylidene difluoride membranes, which were then blocked with skim milk for 1 h and incubated with primary antibodies (1:1000 dilution), followed by incubation with secondary antibody (1:1000 dilution) at room temperature for 1 h. Blots were detected using an enhanced chemiluminescence kit (Vazyme) and analyzed by a Western blot imaging system (Tanon 5200; Tanon Science & Technology). Image analysis was done with ImageJ (National Institutes of Health).

### Immunofluorescence staining

For cellular immunofluorescence staining, cells incubated with sEVs were fixed with 4% paraformaldehyde (Beyotime), then permeabilized with 0.1% Triton X-100 (Beyotime), and blocked with 5% bovine serum albumin (Beyotime) for 1 h at room temperature. The primary antibody (1:200 dilution) was incubated overnight at 4 °C. For ZO-1 staining, cells were fixed with cold methanol–acetone followed by mild saponin permeabilization. After washing with PBS, the fluorescent secondary antibody (1:1000 dilution) was incubated for 1 h at room temperature. Subsequently, the cells were stained with 4′,6-diamidino-2-phenylindole (Beyotime), mounted with an antifluorescence quenching agent (SouthernBiotech), and then fluorescence imaging was performed using an inverted laser confocal microscope LSM 880 with Airyscan (Zeiss). For tissue immunofluorescence staining, mice with different treatments were anesthetized and perfused with cold PBS and 4% paraformaldehyde. Then, the brains were soaked in a constant gradient dehydration solution with sucrose and embedded with optimal cutting temperature compound (SAKURA Tissue-Tek) for 30 min at −20 °C. The brains were sliced (25 μm in thickness) with a freezing microtome (Leica Microsystems). The brain sections were blocked with 5% normal horse serum and 0.3% Triton X-100 in PBS for 1 h at room temperature. After washing, the sections were incubated with the primary antibodies (1:200 dilution) overnight at 4°C, and the corresponding fluorescent secondary antibodies (1:1000 dilution) were incubated with the sections. After staining with 4′,6-diamidino-2-phenylindole, the sections were imaged with Olympus VS200 (Olympus) and LSM 880 with Airyscan. Image analysis was performed using OlyVIA 4.1 software (Olympus), Zeiss ZEN Blue 3.1 software (Zeiss), and ImageJ.

### *In vivo* fluorescence imaging

sEVs carrying Cy5-labeled siRNAs were injected *via* the tail vein. One-hour postinjection, brain tissues from sEV-treated mice were excised and subjected to fluorescence imaging using an *in vivo* imaging system (IVIS Lumina XR system I; PerkinElmer). For fluorescence quantification, identical regions of interest were drawn across all samples. Radiance values were determined using IVIS software Living Image version 4.2 (PerkinElmer).

### Statistics

Details on the statistics are shown in the figure legends. All data are presented as the mean ± SEM from at least three independent experiments. All statistical analyses were performed using commercially available software (GraphPad Prism 9; GraphPad Software, Inc). Multiple group comparisons were analyzed by using one-way or two-way ANOVA followed by Tukey’s multiple comparison test. Significance was assumed at ∗*p* < 0.05; ∗∗*p* < 0.01; and ∗∗∗*p* < 0.001.

## Data availability

The raw data supporting the conclusion of this article will be made available by the authors upon reasonable request.

## Supporting information

This article contains supporting information.

## Conflict of interest

The authors declare that they have no conflicts of interest with the contents of this article.
