# Supplementary material for: Selective peptide–guided transcytosis enhances extracellular vesicle–mediated siRNA delivery across the blood–brain barrier
Source: J Biol Chem. 2025 Nov 13;302(1):110942. doi: 10.1016/j.jbc.2025.110942 (PMC12753227; doi:10.1016/j.jbc.2025.110942)
Supplement: Supporting information [file mmc1.pdf]

Selective Peptide-Guided Transcytosis Enhances Extracellular Vesicle-Mediated siRNA Delivery Across the Blood-Brain Barrier.

Jingwen Fang[1, 2, #], Lingzhu Zhang[1, 2, #], Ye Wang[1, 2], Menghan Chen[1, 2], Yanqiu He[1, 2], Chen-Yu Zhang[1, 2], Yujing Zhang[1, 2], Xiaohong Jiang[1, 2, *], Jing Li[1, 2, *].

Materials list:
Supplementary Figures (1-6)
Supplementary Table (1)

Supplementary Figures

Supplementary Figure 1

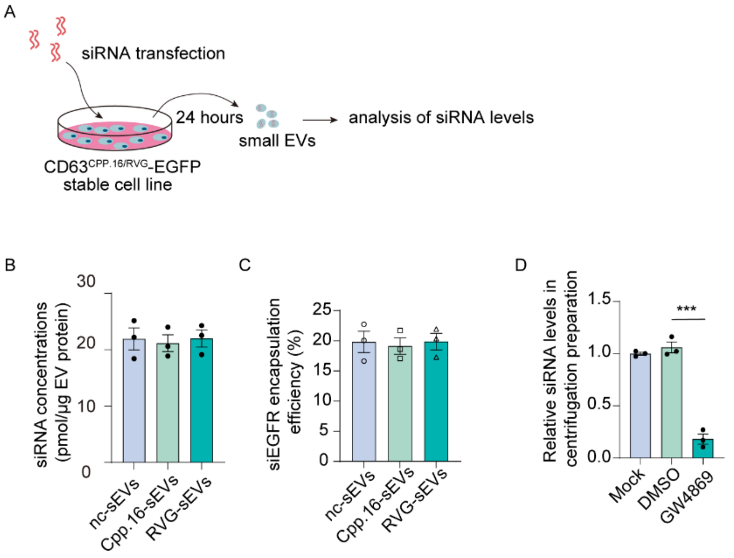

Supplementary Figure 1. siRNA Loading into Peptide-Modified sEVs. (A) Schematic illustration of generation of three types of siRNA-loaded engineered sEVs. (B) Quantitative RT-PCR analysis of siRNA levels in sEVs (*n* = 3 per group). (C) siRNA encapsulation efficiency, defined as the ratio of encapsulated siRNA levels to the total transfected siRNAs (*n* = 3 per group). (D) Quantitative RT-PCR analysis of siRNA levels in centrifugation preparation from cells treated with DMSO or GW4869 (*n* = 3 per group). Data are presented as the mean ± SEM. *p-values* were determined using one-way ANOVA followed by Tukey's multiple comparison test in B and D, *$p < 0.05$, ***$p < 0.001$.

Supplementary Figure 2

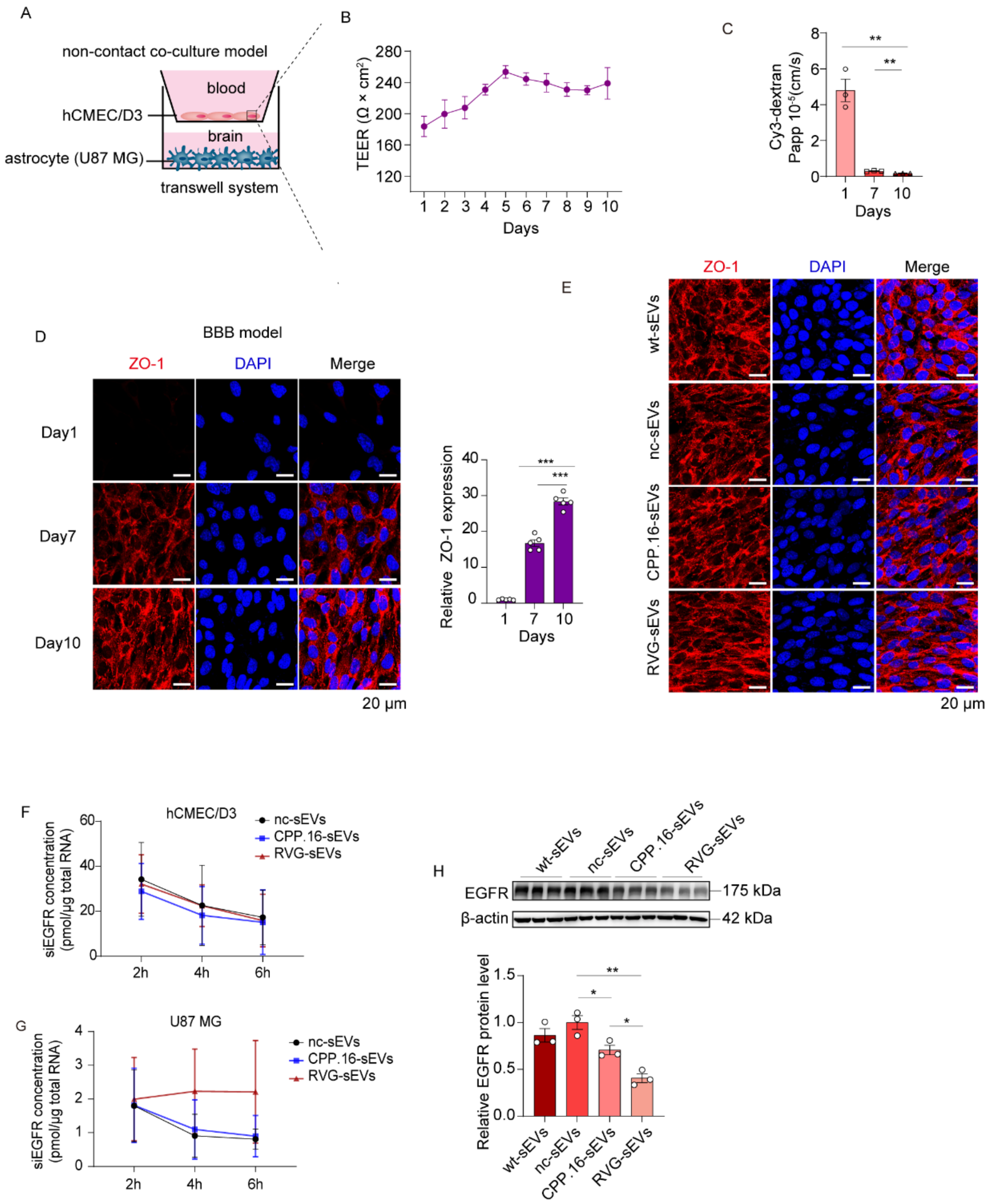

Supplementary Figure 2. Validation of the In Vitro BBB Model. (A) Schematic illustration of the BBB model. (B) TEER values of hCMEC/D3 cells co-cultured with U87MG cells over 10 days ($n = 6$ per group). (C) Dextran permeability of the BBB model ($n = 3$ per group). (D) Fluorescence images and quantitative analysis of the tight-junction protein ZO-1 (red) in hCMEC/D3 cells. Nuclei: DAPI (blue). Scale bar: 20 μm. ($n = 5$ per group). (E) Fluorescence images and quantitative analysis of ZO-1 in hCMEC/D3 cells upon the treatment of sEVs ($n = 3$ per group). (F-G) Quantitative RT-PCR analysis of siRNA levels in hCMEC/D3 (F) and U87MG (G) cells at 2, 4, and 6 hours post-incubation. ($n = 9$ per group). (H) Western blot analysis of EGFR levels in U87MG cells in the BBB model. Data are presented as the mean ± SEM. *p-values* were determined using one-way ANOVA followed by Tukey's multiple comparison test in C, D and H, *$p < 0.05$, **$p < 0.01$, ***$p < 0.001$.

Uncropped western blot images for (H) are shown in **Figure S6**.

Supplementary Figure 3

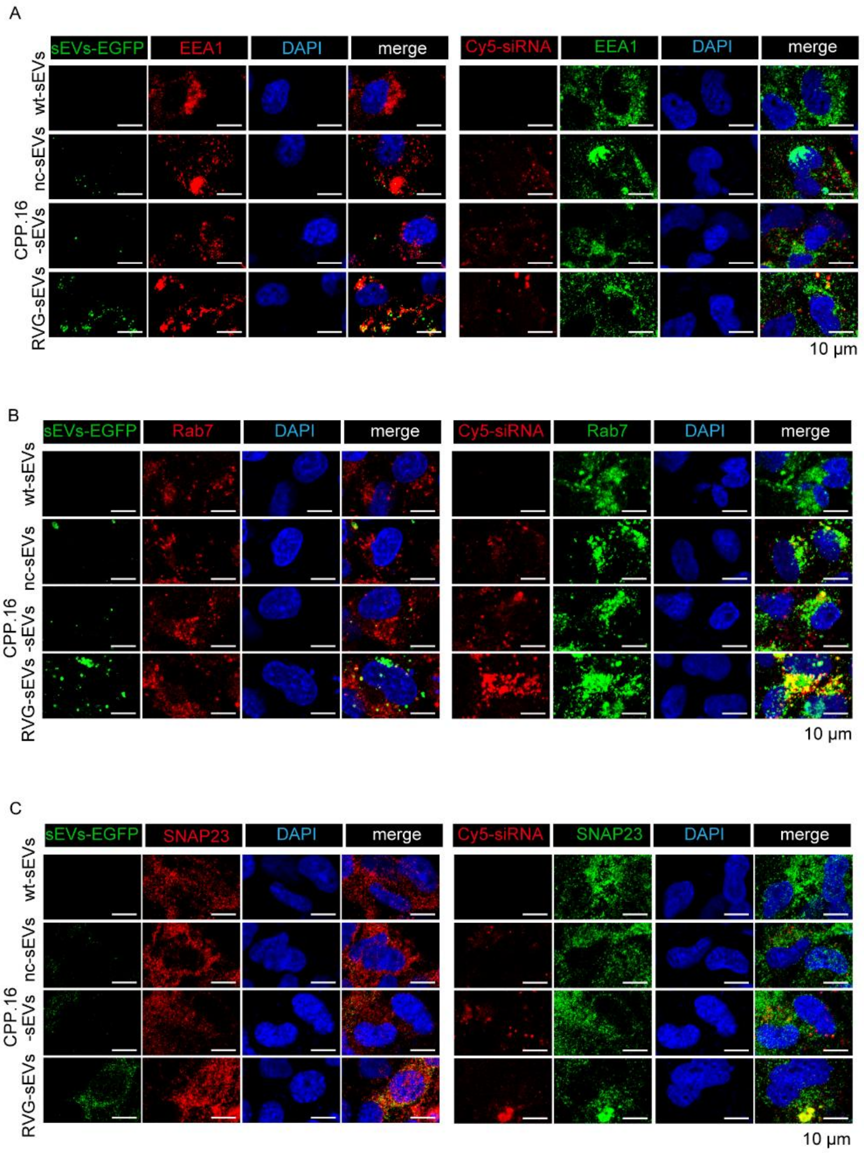

Supplementary Figure 3. Transcytosis of Peptide-Modified sEVs. (A-C) Fluorescence images showing colocalization of sEVs or Cy-5-siRNAs with early endosome marker EEA1 (A), late endosome marker Rab7 (B), and SNAP23 (C). Nuclei: DAPI (blue). Scale bar: 10 μm.

Supplementary Figure 4

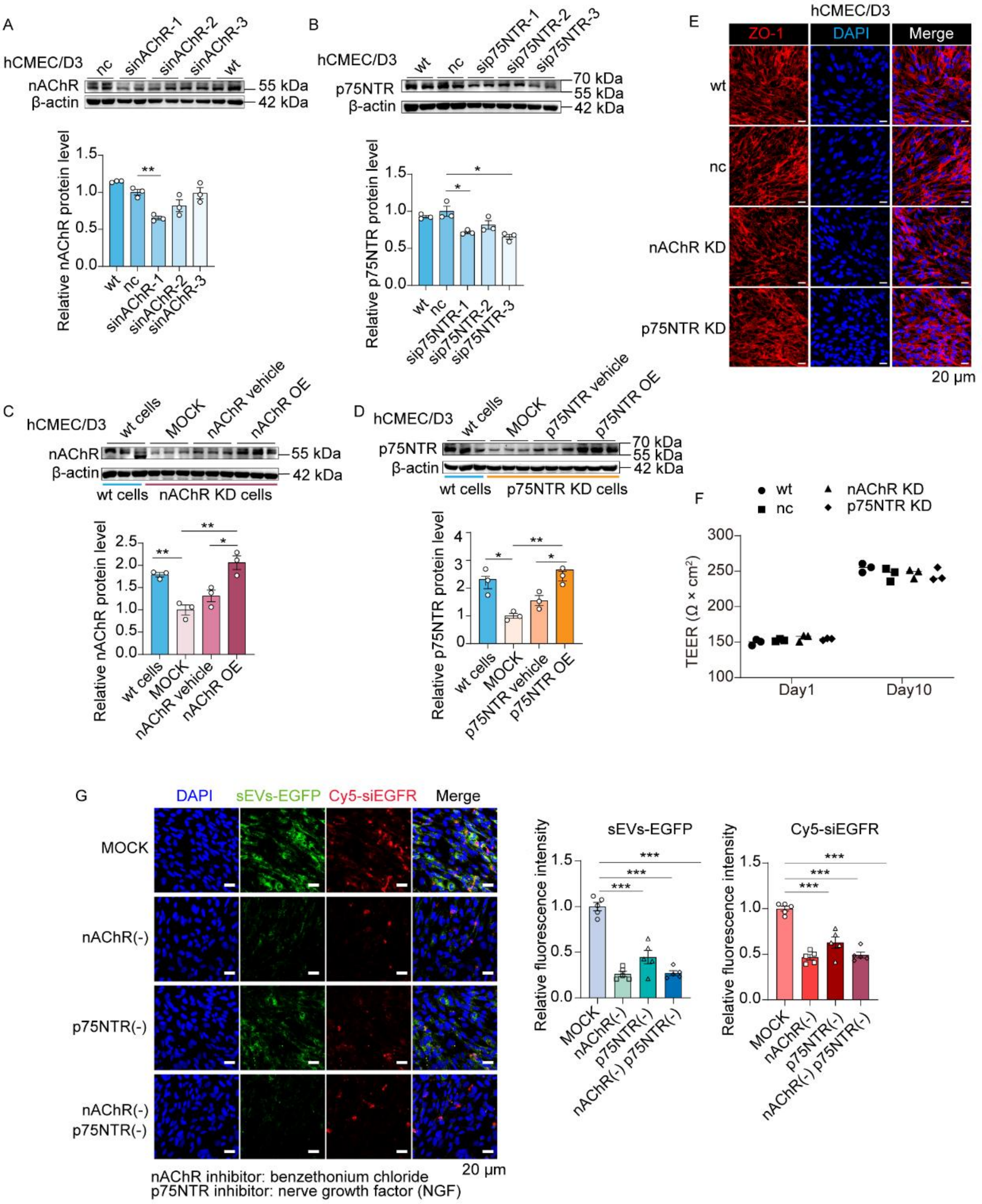

Supplementary Figure 4. Evaluation of receptor-mediated transcytosis. (A) Western blot analysis of nAChR levels in hCMEC/D3 cells transfected with nAChR siRNAs (n = 3 each group). (B) Western blot analysis of p75NTR levels in hCMEC/D3 cells transfected with p75NTR siRNAs (n = 3 each group). (C) Western blot analysis of nAChR levels in wild-type hCMEC/D3 cells, nAChR KD cells, nAChR KD cells transfected with vehicle plasmid, or nAChR KD cells transfected with an nAChR-overexpressing plasmid (n = 3 per group). (D) Western blot analysis of p75NTR levels in wild-type hCMEC/D3 cells, p75NTR KD cells, p75NTR KD cells transfected with vehicle plasmid, or p75NTR KD cells transfected with p75NTR -overexpressing plasmid (n = 3 per group). (E) Fluorescence images of ZO-1 in receptor KD hCMEC/D3 cells. (F) TEER values of receptor KD hCMEC/D3 cells co-cultured with U87MG cells at day 10 (n = 3 per group). (G) Fluorescence images of sEVs-EGFP (green) and Cy5-siEGFR (red) in

hCMEC/D3cells treated with benzethonium chloride (nAChR antagonist, 42 µM, 60 minutes) or nerve growth factor (p75NTR inhibitor, 50 ng/mL, 30 minutes). Nuclei: DAPI (blue). Scale bar: 20 µm. And fluorescence intensity quantification of sEVs-EGFP and Cy5-siEGFR normalized to DAPI. (*n* = 5 per group). Data are presented as the mean ± SEM. *p-values* were determined using one-way ANOVA followed by Tukey's multiple comparison test in A-D, and G, *$p < 0.05$, **$p < 0.01$, ***$p < 0.001$. Uncropped western blot images for (A-D) are shown in **Figure S6**.

Supplementary Figure 5

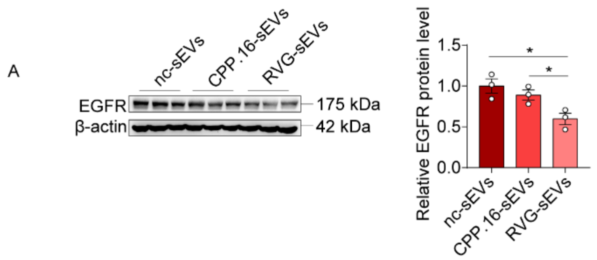

Supplementary Figure 5. RVG-sEVs Achieve Targeted In Vivo Brain Delivery. (A) Western blot analysis of EGFR levels in mouse brain injected with sEVs (*n* = 3 per group). Data are presented as the mean ± SEM. *p-values* were determined using one-way ANOVA followed by Tukey's multiple comparison test in A, *$p < 0.05$.
Uncropped western blot images for (A) are shown in **Figure S6**.

Supplementary Figure 6

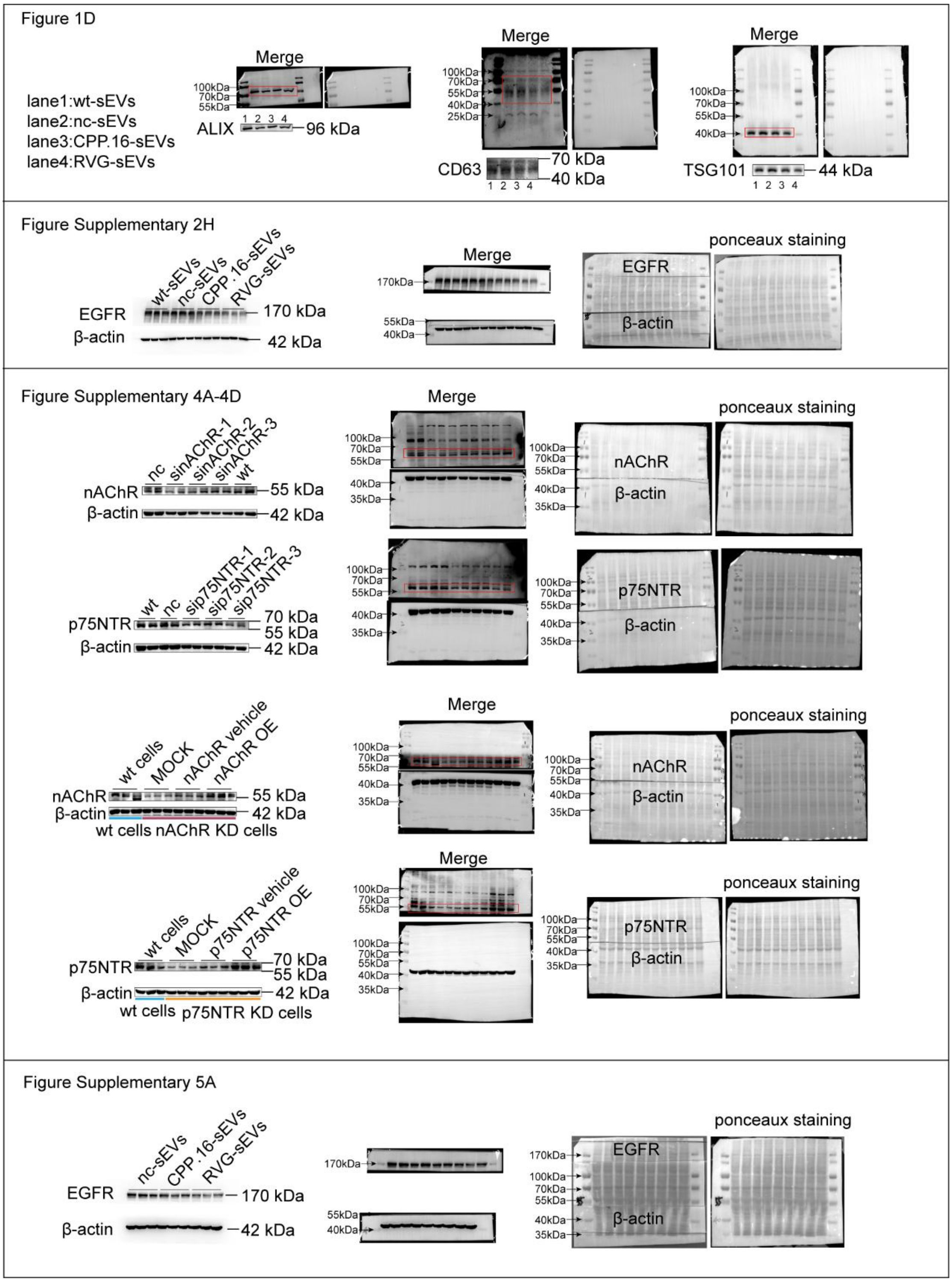

Supplementary Figure 6. Uncropped western blot images for Figure 1D, S2H, S4A-S4D and S5A.

Supplementary Table 1:

EGFR siRNA (mmu):	5'-TGTGGCTTCTCTTAACTCCT-3';
EGFR siRNA (hsa):	5'-ATACCTATTCCGTTACACACT-3';
nAChR siRNA-1 (hsa):	5'-GCAACCACTCACCGTCTACTT-3';
nAChR siRNA-2 (hsa):	5'-GAAACCAGACATTCTTCTCTA-3';
nAChR siRNA-3 (hsa):	5'-AATGGGACCTAGTGGGAATCC-3';
p75NTR siRNA-1 (hsa):	5'-AACAAGACCTCATAGCCAGCA-3';
p75NTR siRNA-2 (hsa)	5'-GGAGGAGGTGGAGAAGCTTCT-3';
p75NTR siRNA-3 (hsa)	5'-AAGCTTCTCAACGGCTCTGCG-3'

Sequences with underlines are used for lentivirus packaging.
